# Immunoreactive insulin stability in horses at risk of insulin dysregulation

**DOI:** 10.1111/jvim.15629

**Published:** 2019-10-16

**Authors:** Dakota H. Leschke, Genevieve S. Muir, Jack K. Hodgson, Mitchell Coyle, Remona Horn, François‐René Bertin

**Affiliations:** ^1^ School of Veterinary Science The University of Queensland Gatton Queensland Australia; ^2^ School of Agriculture and Food Sciences The University of Queensland Gatton Queensland Australia

**Keywords:** chemiluminescent assay, diagnostic testing, endocrinology, equine metabolic syndrome, laminitis, obesity

## Abstract

**Background:**

Diseases associated with insulin dysregulation (ID), such as equine metabolic syndrome and pituitary pars intermedia dysfunction, are of interest to practitioners because of their association with laminitis. Accurate insulin concentration assessment is critical in diagnosing and managing these diseases.

**Hypothesis/Objectives:**

To determine the effect of time, temperature, and collection tube type on insulin concentrations in horses at risk of ID.

**Animals:**

Eight adult horses with body condition score >6/9.

**Methods:**

In this prospective study, subjects underwent an infeed oral glucose test 2 hours before blood collection. Blood samples were divided into ethylenediaminetetraacetic acid, heparinized, or serum tubes and stored at 4 or 20°C. Tubes were centrifuged and analyzed for insulin by a chemiluminescent assay over 8 days. Changes in insulin concentrations were compared with a linear mixed effects model.

**Results:**

An overall effect of time, tube type and temperature was identified (*P* = .01, *P* = 0.001, and *P* = 0.001, respectively). Serum and heparinized samples had similar concentrations for 3 days at 20°C and 8 days at 4°C; however, after 3 days at 20°C, heparinized samples had significantly higher insulin concentrations (*P* = .004, *P* = .03, and *P* = .03 on consecutive days). Ethylenediaminetetraacetic acid samples had significantly lower insulin concentrations regardless of time and temperature (*P* = .001 for all comparisons).

**Conclusions and Clinical Importance:**

These results suggest an ideal protocol to determine insulin concentrations involves using serum or heparinized samples with analysis occurring within 3 days at 20°C or 8 days at 4°C.

**Abbreviations**
BCS: body condition score
CLIA: chemiluminescence immunoassay
EDTA: ethylenediaminetetraacetic acid
EMS: equine metabolic syndrome
ID: insulin dysregulation
OGT: oral glucose test
OST: oral sugar test
RIA: radioimmunoassay

## 1 INTRODUCTION

An aging equine population and increased prevalence of obesity together with a better understanding of equine endocrine disorders have led to a rise in the recognition of endocrinopathies in horses.^1^, ^2^, ^3^ Equine metabolic syndrome (EMS) and pituitary pars intermedia dysfunction are of particular importance because of their association with laminitis.^4^, ^5^ A fundamental feature of EMS is insulin dysregulation (ID) which describes alterations in insulin metabolism, namely hyperinsulinaemia, excessive insulin secretion after a carbohydrate challenge, peripheral tissue insulin resistance, or a combination of those elements.^6^ As excessive insulin concentrations have been associated with an increased risk of developing laminitis, an accurate diagnosis of ID is therefore critical for ensuring optimal patient health and welfare outcomes.^7^, ^8^

Diagnosis of ID involves demonstration of resting (basal) hyperinsulinaemia, demonstration of excessive insulin response to an oral carbohydrate stimulation, and/or demonstration of peripheral tissue insulin resistance.^6^ Convenient dynamic tests to detect peripheral tissue insulin resistance and enteroinsular axis activity have been developed for use in practice. The 2‐step insulin sensitivity test, measures glucose in response to an IV insulin challenge whereas the oral glucose test (OGT) and the oral sugar test (OST) measure insulin after an oral glucose challenge.^9^, ^10^ The OGT and the OST, which are equivalent to identify horses with ID, are frequently used in practice and make insulin a commonly measured analyte.^5^, ^10^

In remote equine practice, accuracy of laboratory results can be affected by prolonged time to analysis because of considerable distances between horses and testing laboratories. The effects of handling on sample stability for multiple analytes have been researched in horses.^11^, ^12^ However, information regarding the most appropriate techniques and protocols for maintaining stability of immunoreactive insulin in horses is poorly described. Human studies have identified improved immunoreactive insulin stability in whole blood stored in ethylenediaminetetraacetic acid (EDTA) tubes; as such EDTA has replaced serum as the conventional storage medium for insulin analysis in people.^13^ Serum is still the mainstay for insulin quantification in equine practice; however, it remains to be seen whether the potential benefits of EDTA are compatible with equine endocrinological testing.^14^

Previous studies have concluded that storage of equine serum or plasma samples at room temperature over 3 days had minimal effect on immunoreactive insulin concentration when analyzed via a radioimmunoassay (RIA) technique.^14^ However, with the RIA no longer available, practitioners and laboratories have since relied on a variety of assays, with the chemiluminescence immunoassay (CLIA) being the most common.^15^ The RIA and the CLIA use different antibodies to detect insulin and the type of assay used greatly impact results when measuring immunoreactive insulin concentrations.^16^, ^17^ Therefore, results found previously using the RIA might not be as relevant to practitioners now utilizing the available CLIA as each assay measures different antigens with possibly different stabilities.

Inconsistencies between assays and a lack of in‐depth research leave little information available for equine practitioners on the best methods available to ensure stability of equine insulin. The aim of the study was therefore to determine the effects of sample storage conditions (time, temperature, and tube type) on measurements of immunoreactive insulin concentration using the CLIA in a clinically relevant context using horses at risk of ID.

## 2 MATERIALS AND METHODS

### 2.1 Horses

Eight adult horses (4 geldings and 4 mares) of varying breeds (3 Warmbloods, 2 Standardbreds, 2 Australian Stock Horses, and 1 Quarter Horse) were selected from the institution's equine research herd. Specifically, inclusion criteria for horses consisted of a body condition score (BCS) ≥6/9 so as to increase the likelihood of selecting horses with ID.^4^ Horses averaged an age of 15 ± 4 years with a mean body weight of 583 ± 40 kg, and a median BCS of 7/9 (6‐8).^18^ Each animal was deemed healthy before the study by physical examination. Ethical approval was obtained from the institutional animal care and ethical use committee.

### 2.2 Design

Horses were stabled and fasted for 10 hours, with free access to water before testing.^19^ After collection of baseline blood samples, horses were fed dextrose powder (Dextrose powder, Gatton Home Brew, Camping & Fishing Supplies, Gatton, Australia) at 1 g/kg body combined with 500 g of a form of micronized sweet feed (HyGain Feeds Pty Ltd, PO Box 199, Victoria, Australia) and 500 g of lucerne chaff. A second blood sample was collected 2 hours later for analysis.

At the time of collection of the second sample, a multi‐sample 18‐gauge vacuum collection needle and a disposable tube holder (BD, Belliver Industrial Estate, Plymouth, UK) were used to collect a total of 36 samples from each horse via direct left jugular venipuncture. Blood was collected into lithium heparin, EDTA (BD, Belliver Industrial Estate) and silicate‐containing serum (BD, Belliver Industrial Estate) tubes. To reduce variation in results, samples were taken within a 5‐minute time period, and tube selection randomized.^20^

For each horse, 12 serum, 12 heparin, and 12 EDTA tubes were obtained and stored unseparated at 4°C or at 20°C for up to 8 days. Samples were centrifuged at 3000*g* and analyzed daily over the course of 5 days, with Day 1 being the day of collection, and with an additional analysis conducted on Day 8. On days of analysis, a 4 and 20°C tube from each tube type was randomly selected for each horse and, after centrifugation, the resulting serum or plasma sample was assessed for hemolysis. Hemolysis was graded visually on a scale from 0 to 3, with 0 being non‐hemolyzed, transparent serum or plasma, and 3 being completely hemolyzed, dark red fluid (Supplementary Figure S1). The serum or plasma was pipetted using a 1‐mL plastic disposable pipette into 1.5‐mL aliquot tubes (Volume 1.5 mL Graduated Microcentrifuge Tube, QSP Scientific Plastics, San Diego, California). Samples were then analyzed using a commercially available CLIA (Immulite 1000 Chemiluminescent Assay, Siemens, Bayswater, Victoria, Australia) to determine serum or plasma immunoreactive insulin concentrations.

In order to determine the inter‐ and intra‐assay coefficient of variation of the CLIA in serum, heparinized, and EDTA samples, additional blood samples were collected from 8 additional horses with insulin concentrations ranging from 3.0 to 197.0 μIU/mL. Samples were processed at 4°C as described above and run twice on kits with the same lot number (intra‐assay coefficient of variation) and an additional time on kits with a different lot number (inter‐assay coefficient of variation).

### 2.3 Data analysis

A Shapiro‐Wilk test was conducted to assess for a normal distribution. Normally distributed data for age and weight were presented as mean ± SD, whereas BCS, as an ordinal variable, was presented as median (range). A linear mixed effects model was used to determine changes in immunoreactive insulin concentrations. The variables “time,” “temperature,” “tube,” and “hemolysis” were included as fixed effects, whereas “horse” was included as a random effect. Transformation of the immunoreactive insulin concentrations into percentage of baseline was required to satisfy residual normality; the baseline used for comparison among individual horses was the value collected from serum samples stored at 4°C on Day 1, as this reading most closely resembles the patient's true insulin concentration at the time of sampling.^14^ Then a 1‐way repeated measures ANOVA was performed to detect conditions significantly different from Day 1, 4°C, and serum sample. Statistical analysis was carried out using commercial statistical software (Prism, GraphPad Software, Inc. La Jolla, California; IBM SPSS Statistics 24, IBM Corp. Armonk, New York). A *P*‐value <.05 was considered significant.

## 3 RESULTS

Overall, there was a significant effect of temperature (*P* = .001), collection tube (*P* = .001), and the time to centrifugation (*P* = .01) on equine immunoreactive insulin concentration.

For serum samples, the intra‐assay coefficient of variation was 6.5% and the inter‐assay coefficient of variation was 7.9%. For heparinized samples, the intra‐assay coefficient of variation was 6.1% and the inter‐assay coefficient of variation was 12.5%. For EDTA samples, the intra‐assay coefficient of variation was 4.0% and the inter‐assay coefficient of variation was 14.4%.

**Figure 1:**
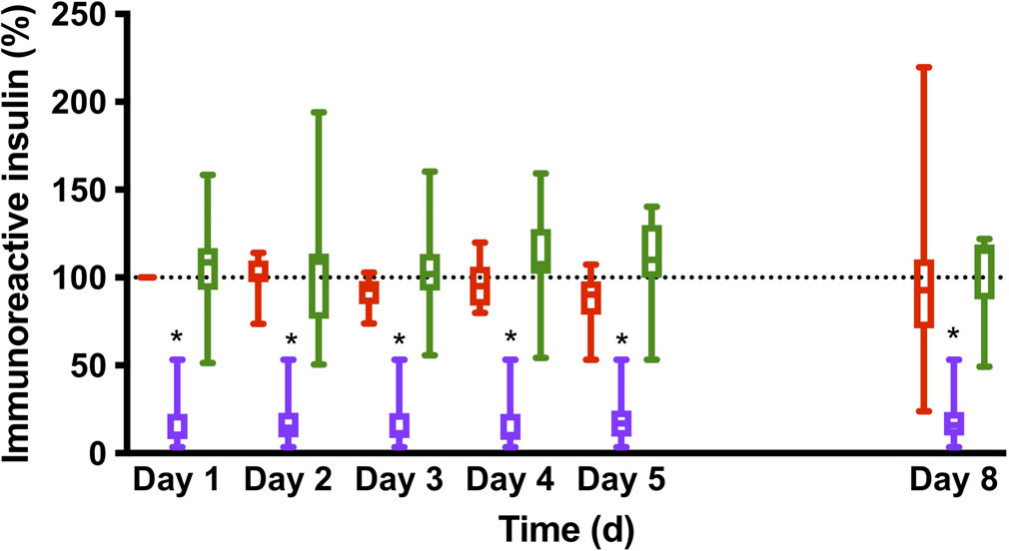
Changes in concentrations of immunoreactive insulin (as percentage) over time in samples collected in silicate‐containing tubes (red), lithium heparin tubes (green), or ethylenediaminetetraacetic acid (EDTA) tubes (purple) and stored at 4°C. Data are represented as whisker plots showing the median, the first and third quartile as well as the minimal and maximal values (n = 8). *P < .05 versus serum

At 4°C, there was no significant difference in immunoreactive insulin concentrations between serum and heparinized samples at any time point and the median concentrations were close to their assay coefficients of variation. In contrast, immunoreactive insulin concentrations in the EDTA samples were significantly lower than in serum samples from Day 1 to Day 8 (*P* = .001 for all comparisons, Figure 1 and Table 1) with median immunoreactive insulin concentrations in EDTA samples ranging from 10.5% to 17.0% of the immunoreactive insulin concentrations in serum samples.

**Table 1:** Percentage of baseline insulin in serum, heparinized, or ethylenediaminetetraacetic acid (EDTA) samples kept at 4 or 20°C for up to 8 days from 8 obese horses that underwent an oral glucose test

	4°C			20°C		
	Serum	Heparin	EDTA	Serum	Heparin	EDTA
Day 1	100.0	108.6 (51.4‐158.5)	10.6 (3.4‐53.2)*	95.5 (60.4‐116.0)	97.5 (36.0‐130.2)	10.9 (3.4‐53.2)*
Day 2	100.6 (73.6‐114.0)	109.1 (50.5‐194.0)	14.5 (3.4‐53.2)*	91.1 (48.3‐118.4)	119.9 (47.1‐137.7)	15.3 (3.4‐53.2)*
Day 3	93.7 (73.9‐102.8)	102.0 (55.7‐160.4)	11.4 (3.4‐53.2)*	92.9 (58.8‐108.7)	103.5 (47.4‐147.5)	14.0 (3.4‐53.2)*
Day 4	95.1 (79.9‐119.9)	107.6 (54.3‐159.2)	10.4 (3.4‐53.2)*	90.6 (53.2‐100.7)	113.8 (40.0‐144.9)*	10.5 (3.4‐53.2)*
Day 5	90.4 (53.2‐107.3)	110.2 (53.3‐140.4)	17.0 (3.4‐53.2)*	85.2 (51.0‐109.9)	108.5 (32.1‐137.7)*	12.9 (3.4‐53.2)*
Day 8	92.7 (23.9‐219.6)	115.4 (49.3‐121.9)	16.0 (3.4‐53.2)*	54.6 (41.2‐76.9)** ^,^ ***	81.7 (34.9‐129.8)* ^,^ ***	8.4 (3.4‐53.2)* ^,^ ***

*P < .05 versus serum; **P < .05 versus Day 1; ***P < .05 versus 4°C.

**Figure 2:**
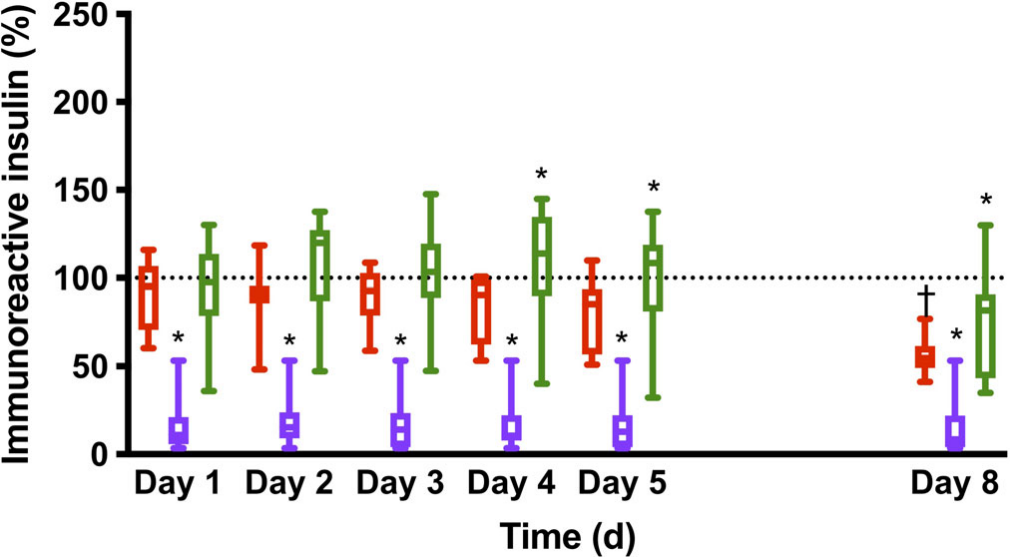
Changes in concentrations of immunoreactive insulin (as percentage) over time in samples collected in silicate‐containing tubes (red), lithium heparin tubes (green), or ethylenediaminetetraacetic acid (EDTA) tubes (purple) and stored at 20°C. Data are represented as whisker plots showing the median, the first and third quartile as well as the minimal and maximal values (n = 8). *P < .05 versus serum and ^†^ *P* < .05 versus Day 0

At 20°C, there was no significant difference in immunoreactive insulin concentrations between serum and heparinized samples until Day 3; however, there was more variability and median concentrations were often not within their assay coefficients of variation. After Day 4, immunoreactive insulin concentrations in heparinized samples were significantly higher than in serum samples (*P* = .004 on Day 4, *P* = .03 on Day 5, and *P* = .03 on Day 8, Table 1). Immunoreactive insulin concentrations in EDTA samples were also significantly lower than in serum samples from Day 1 to Day 8 (*P* = .001 for all comparisons, Figure 2 and Table 1) with median immunoreactive insulin concentrations in EDTA samples ranging from 8.4% to 15.3% of the immunoreactive insulin concentrations in serum samples. In all sample types, immunoreactive insulin concentrations were significantly lower on Day 8 at 20°C than on Day 8 at 4°C (*P* = .009 for serum, *P* = .01 for heparin, and *P* = .04 for EDTA, Table 1) and in serum samples at 20°C, immunoreactive insulin concentrations were significantly lower on Day 8 than on Day 1 (*P* = .001, Table 1).

## 4 DISCUSSION

This study investigated the effect of handling protocol on the concentration of immunoreactive insulin in horses at risk of ID. Our findings indicate that immunoreactive insulin concentrations in both serum and heparinized plasma remain stable up to 8 days when stored at 4°C, and up to 3 days when stored at 20°C. In contrast, immunoreactive insulin in EDTA samples measurements remained consistently lower than in serum and heparinized samples regardless of storage temperature or time at which analysis was conducted. Our results are in agreement with previous equine studies using the RIA and provide practitioners using the CLIA with confidence that a longer interval between sample collection and analysis can be utilized, reducing time and laboratory deadline pressures on clinicians.^14^

Type of blood tube was the main factor of variability between samples and is also shown by the increased assay coefficients of variation in heparinized and EDTA samples. Immunoreactive insulin concentrations from EDTA samples were consistently lower than immunoreactive insulin concentrations from serum or heparinized samples, regardless of storage temperature or time at which the samples were analyzed. Multiple studies have concluded that samples collected and stored in EDTA tubes have no effect on insulin degradation.^13^, ^21^ This suggests that the low values and the larger variation are likely related to the assay used, not specifically to EDTA. Unlike samples used to determine ACTH concentrations, the manufacturer does not recommend analysis of EDTA samples with the insulin CLIA, further substantiating this point. Immunoreactive insulin detected using this method from EDTA samples is inaccurate, variable, and as low as 10% of the concentration of immunoreactive insulin detected in serum samples; to the authors' knowledge, there is no prior evidence of this occurring in equid‐derived samples. However, the fact that insulin is analogous among species would explain how the effect was replicated when measuring equine immunoreactive insulin.^22^ As such, the authors do not recommend the use of EDTA tubes for collection of equine insulin blood samples to be analyzed using this assay.

Immunoreactive insulin concentrations in heparinized plasma were more variable and consistently higher than in serum after Day 4 at 20°C which has been observed previously in human studies.^23^ A larger range of values was also observed in heparinized samples compared to serum. The influence of heparin itself is likely the cause of the overestimation in values, but this interaction has not been thoroughly documented.^23^, ^24^ Although the purpose of this study was not to investigate the ability of the OGT to detect ID in obese horses, these unpredictable increases could potentially lead to false positive cases of ID.

In contrast, serum sample ranges were generally lower and less dispersed, even at 20°C and the intra‐ and inter‐assay coefficients of variation were consistent with other studies.^15^ Therefore, in a clinical setting, serum would be preferred when diagnosing ID as it is more likely to give an accurate insulin concentration, whereas heparinized samples are more likely to show an aberrant insulin concentration and an erroneous diagnosis.

Temperature and time were the other factors associated with variations in immunoreactive insulin concentrations. Although statistically significant, this effect only resulted in median variations greater than the intra‐ and inter‐assay coefficients of variation and erroneous diagnoses on Days 5 and 8. This is consistent with a previous study using the RIA in which no statistically significant nor clinically relevant effect of storing samples at room temperature for 3 days was detected.^14^ In that study, the discrepancies between the changes observed in human studies and the absence of effect observed in equine studies were attributed to study design and differences in posttranslational modifications of the insulin peptide between humans and horses.^25^, ^26^

A statistically significant effect of hemolysis on immunoreactive insulin concentration was not detected in our study. The degree of hemolysis can have a significant impact on the amount of analyte measured in an immunoassay and even minor levels of hemolysis may influence immunoreactive insulin measurements.^27^, ^28^ In this study, very few samples were hemolyzed, which limited our power to detect an effect if 1 were present. Out of the 288 samples, only 48 samples showed minor hemolysis and 13 samples showed moderate hemolysis with the rest of the samples showing none. Regardless of tube type, samples stored at 20°C never showed more than minor levels of hemolysis. Of the samples stored at 4°C, both serum and EDTA tubes had several samples with moderate hemolysis and EDTA more so than serum. In contrast, 13 heparinized samples illustrated minor hemolysis across the entire analysis period. Although hemolysis did not significantly influence the accuracy of results in this study, previous studies have demonstrated that an effect likely exists.^21^, ^28^

In conclusion, storage of serum and heparinized samples at 4°C ensures similar measurements of equine immunoreactive insulin for up to 8 days postsampling. At 20°C, however, serum and heparinized samples only show similar immunoreactive insulin concentrations for 3 days, but after Day 4, immunoreactive insulin concentrations in serum samples decrease significantly and immunoreactive insulin concentrations in heparinized samples are more variable. Samples collected in EDTA tubes provided lower readings regardless of the protocol selected than samples collected in other tubes, and EDTA is therefore not recommended for use. The results of this study provide clinicians with appropriate handling guidelines to ensure optimal measurements of equine immunoreactive insulin before analysis with a CLIA.

## CONFLICT OF INTEREST DECLARATION

François‐René Bertin has consulted for and received funding from Boehringer Ingelheim Pty Ltd for his research.

## OFF‐LABEL ANTIMICROBIAL DECLARATION

Authors declare no off‐label use of antimicrobials.

## INSTITUTIONAL ANIMAL CARE AND USE COMMITTEE (IACUC) OR OTHER APPROVAL DECLARATION

Animal ethics was obtained: SVS481/18.

## HUMAN ETHICS APPROVAL DECLARATION

Authors declare human ethics approval was not needed for this study.

## Supporting information


**Supplementary Figure 1** Representative tubes showing hemolysis scores (from left to right: 0 = no visible hemolysis, 1 = mild visible hemolysis, 2 = moderate visible hemolysis and 3 = severe visible hemolysis).
