# Supplementary figures and images for: Immunoreactive insulin stability in horses at risk of insulin dysregulation
Source: J Vet Intern Med. 2019 Oct 16;33(6):2746–51. doi: 10.1111/jvim.15629 (PMC6872612; doi:10.1111/jvim.15629)

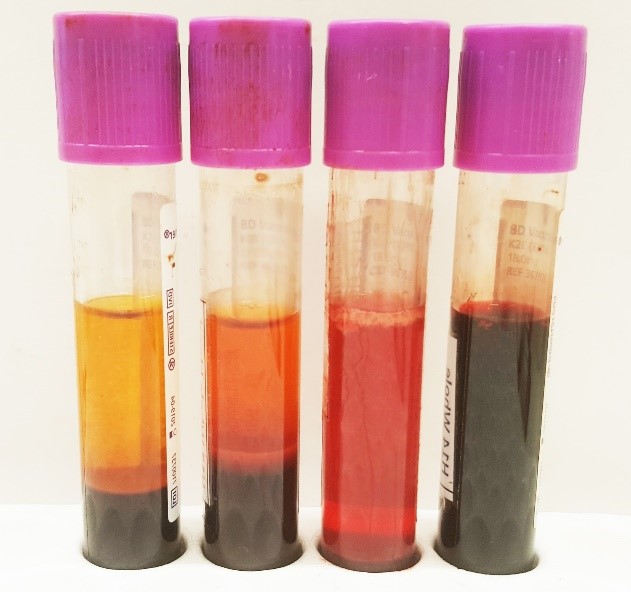

Supplement: Supplementary file 1 — Supplementary Figure 1 Representative tubes showing hemolysis scores (from left to right: 0 = no visible hemolysis, 1 = mild visible hemolysis, 2 = moderate visible hemolysis and 3 = severe visible hemolysis). [file JVIM-33-2746-s001.jpg]
